# Productive Cross-Talk with the Microenvironment: A Critical Step in Ovarian Cancer Metastasis

**DOI:** 10.3390/cancers11101608

**Published:** 2019-10-21

**Authors:** Mohamed A. Abd El Aziz, Komal Agarwal, Subramanyam Dasari, Anirban K. Mitra

**Affiliations:** 1 Medical Sciences Program, Indiana University School of Medicine, Bloomington, IN 47405, USA; mohamad102365@fmed.bu.edu.eg (M.A.A.E.A.); komalbasani@gmail.com (K.A.); sudasari@iu.edu (S.D.); 2 Department of Medical and Molecular Genetics, Indiana University School of Medicine, Indianapolis, IN 46202, USA; 3 Indiana University Melvin and Bren Simon Cancer Center, Indianapolis, IN 46202, USA

**Keywords:** ovarian cancer, microenvironment, metastasis, mesothelial cells, fibroblasts, adipocytes, cross-talk, ECM

## Abstract

Most ovarian cancer patients present with disseminated disease at the time of their diagnosis, which is one of the main reasons for their poor prognosis. Metastasis is a multi-step process and a clear understanding of the mechanism of regulation of these steps remains elusive. Productive reciprocal interactions between the metastasizing ovarian cancer cells and the microenvironment of the metastatic site or the tumor microenvironment play an important role in the successful establishment of metastasis. Much progress has been made in the recent past in our understanding of such interactions and the role of the cellular and acellular components of the microenvironment in establishing the metastatic tumors. This review will outline the role of the microenvironmental components of the ovarian cancer metastatic niche and their role in helping establish the metastatic tumors. Special emphasis will be given to the mesothelial cells, which are the first cells encountered by the cancer cells at the site of metastasis.

## 1. Introduction

Ovarian cancer is the most lethal gynecological cancer and the fifth leading cause of cancer-related deaths among women in the USA [1]. It is estimated that in 2019, about 22,000 women will be diagnosed with ovarian cancer and approximately 1400 will succumb to it in the USA [1]. Most patients are diagnosed with metastatic disease and this causes the poor prognosis [2]. If detected early, the survival rate is significantly higher. However, early stages of ovarian cancer do not show any noteworthy symptoms and as the disease progress, the symptoms are vague and can be falsely attributed to other conditions. In February 2018, the US preventive service task force recommended against a screening program for ovarian cancer in women without any symptoms, if they do not carry any genetic risk factors [1]. This makes it an even bigger challenge to detect ovarian cancer in the early stages. Therefore, a majority of patients will be treated for metastatic disease. Since metastasis remains the least understood aspect of cancer, it is imperative to focus on increasing our knowledge of the mechanisms of regulation of its critical steps, in order to treat patients effectively. Moreover, it is important to take into consideration the various stromal components of the tumor microenvironment, as well as the microenvironment of the metastatic site, in the context of their role in inhibiting or facilitating metastasis [3].

Epithelial ovarian cancer is the most common type, consisting of 90% of the cases [4]. It is further classified into serous, mucinous, endometroid and clear cell carcinoma. Among them, high-grade serous ovarian carcinoma (HGSOC) is the most common and lethal subtype and accounts for about 70% of ovarian cancer-related deaths [4,5]. For a long time, the site of origin of epithelial ovarian cancer was thought to be the ovarian surface epithelium. However, careful analysis of fallopian tube fimbria from breast cancer type 1/2 (BRCA1/2) carriers undergoing bilateral salpingo-oophorectomy, identified serous tubal intraepithelial carcinomas (STICs) present on the fallopian tube fimbria, as the precursors of HGSOC [6]. Subsequently, several groups have established the fallopian tube as the precursor of HGSOC. However, they can also be a site for metastasis in rare circumstances [7]. In most cases, even though they originate as STICs in the fallopian tubes, they are disseminated to the neighboring ovary, where the large primary tumor is formed. 

**Figure 1:**
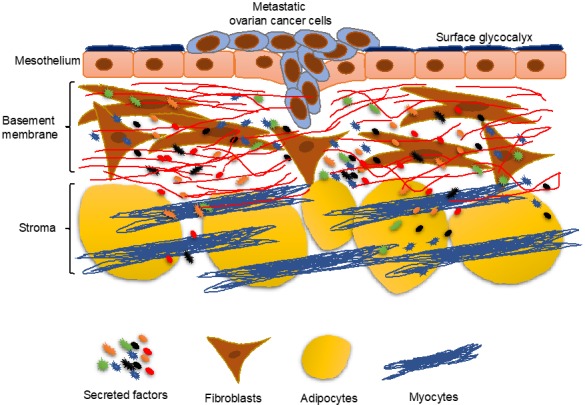
The peritoneal microenvironment encountered by the metastasizing ovarian cancer cells during the initial steps of metastatic colonization.

Ovarian cancer predominantly undergoes a transcoelomic metastasis, where the cancer cells from the primary tumor shed into the peritoneal cavity [8]. The flow of the peritoneal fluid then helps spread them throughout the peritoneal cavity. At this stage, the cancer cells have to survive death due to anoikis as well as immune surveillance. This is usually accomplished by aggregating into spheroids and secreting extracellular matrix proteins to engage the cellular integrins. In addition, the cancer cells have been reported to closely interact with microenvironment cells like cancer-associated fibroblasts (CAFs), to help tide over the conditions while floating in the peritoneal fluid [9]. The cancer cells then attach to the surface of the organs in the peritoneal cavity, most of which are covered by a layer of mesothelium. Thereafter, they have to breach this protective covering and invade into the underlying basement membrane (Figure 1). During this stage, they encounter a new microenvironment, and those that successfully adapt to it eventually colonize the site of metastasis. The metastasizing cancer cells are the most vulnerable at this stage, called metastatic colonization. Therefore, it is considered the rate-limiting step of metastasis. Ovarian cancer can also metastasize through the hematogenous or lymphatic routes, which are less predominant initially but may be responsible at late stages when the disease spreads beyond the peritoneal cavity [8,10]. This review will focus on metastatic colonization, taking into consideration the productive reciprocal interactions with the microenvironment, especially the mesothelium that they encounter first, necessary for the successful establishment of the metastatic tumors. The important secreted factors involved in the cross-talk are listed in Table 1.

## 2. Overview of the Mesothelium

The peritoneum is derived from mesoderm and is composed of two layers: Parietal and visceral layers. The parietal layer lines the abdominal and pelvic walls while the visceral layer surrounds the organs. The space between the parietal and visceral layer is called the peritoneal cavity. This cavity normally contains a small amount of fluid composed of water, electrolytes, solutes, proteins and cells. This fluid normally passes through the mesothelial layer to the lymphatic system. The origin of this fluid is ultrafiltration from the capillaries, which is then drained into the lymphatic system [11]. Histologically, the peritoneal layers consist of a continuous, single layer of squamous cells called mesothelial cells, with an underlying basement membrane. The mesothelial cells are tightly adherent to each other by intercellular junctions and have microvilli on their surface [11]. The mesothelial cells are polar with their apical surface facing the peritoneal cavity and their basal surface attached to the basement membrane. The basement membrane consists of extracellular matrix proteins and embedded fibroblasts. Underneath the basement membrane is the tissue stroma, containing cellular components such as fibroblasts and macrophages and non-cellular components like collagen and glycoproteins [11]. 

The mesothelium not only forms a protective covering, it also serves as a semipermeable membrane. The mesothelial cells help in the passage of intraperitoneal fluid to the lymphatic system and the passage of other substances in and out of the peritoneal cavity [12]. They secrete phospholipids and have a surface glycocalyx that helps generate a non-adhesive, protective surface and facilitate intraperitoneal organ movements during peristalsis [13]. The mesothelium also has an interactive role with its surrounding microenvironment during inflammation and cancer [13]. The mesothelial cells recognize microbial pathogens that might invade the peritoneal cavity. This function is facilitated by the expression of Toll-like receptors, nucleotide-binding oligomerization domain–like receptors, retinoic acid-inducible gene-I-like receptors (RIG-I–like), and C-type lectin-like receptors on the mesothelial cells [14]. The cytokines, growth factors, and extra cellular matrix (ECM) molecules secreted by the mesothelial cells help in coordinating inflammatory responses and tissue repair [15]. Mesothelial cells secrete transforming growth factor (TGF), platelet-derived growth factor (PDGF), fibroblast growth factor (FGF), hepatocyte growth factor (HGF), keratinocyte growth factor, and epithelial growth factors (EGFs) [12,16]. These can promote autocrine as well as paracrine effects. An interesting property of the mesothelium is their mechanism of repair, in case of wounding. Mesothelial cells can migrate from the edges to cover the wound, but also can detach from opposing surfaces and distant sites to settle on the wound surface to initiate repair [17]. In case of ovarian cancer, the mesothelium serves as the first line of defense against the metastasizing cancer cells [18,19].

## 3. Priming the “Soil”

Stephen Paget was the first to suggest that metastatic homing of the cancer cells is not a random process [20]. Interactions between cancer cells (the seed) and the metastatic site (the soil) regulate it. Metastasis is successful only when the target metastatic site has the appropriate properties to accept the message (molecular invitation) from the cancer cells [20,21]. In 1981, Hart et al. Suggested that sarcoma from ovarian origin always metastasized to the abdominal organs whatever the rout of its injection. When these ovarian cancer cells were injected intravenously, they were arrested in the lungs and then slowly detached, recirculated, and were arrested in liver were they subsequently developed into tumor nodules, which supported the soil and seed theory [22]. Similarly, ovarian cancer cells homed in on the ovaries or omentum in mice [10,23]. In order to build an effective soil or premetastatic niche (PMN), cancer cells secrete exosomes—extracellular vesicles containing DNA, messenger RNA (mRNA), microRNA (miRNA), proteins, lipids and small metabolites etc. These exosomes have been shown to carry the message to the PMNs and direct them to prepare a microenvironment suitable for the future metastatic cells [24,25]. Since in ovarian cancer the primary tumor is present in the peritoneal cavity, exosomes or secreted factors can potentially accumulate in relatively high localized concentrations in the peritoneal fluid. As a result, these secreted factors may influence the peritoneal microenvironment and prime them to accept metastasizing ovarian cancer cells. While several studies have been conducted on the priming of the metastatic niche in other cancers, future studies need to focus on the role of priming of the peritoneal microenvironment by ovarian cancer [26,27,28,29].

## 4. Cancer Cells Induce Changes in the Mesothelium

### 4.1. Fibronectin Secretion

Mesothelial cells are considered the first line of defense against ovarian cancer metastatic colonization in the peritoneum. Ovarian cancer cells attach more efficiently to the extracellular matrix and fibroblast than to mesothelial cells [18]. However, during the early stages of cancer metastasis, the induction of epithelial to mesenchymal transitions (EMT) causes a loss of the cell-cell adherence because of the loss of E-cadherin. This is associated with increased expression of α5-integrin through the epidermal growth factor receptor/focal adhesion kinase/mitogen-activated protein kinase (EGFR/FAK/MAPK) pathway, which forms a heterodimer with β1-integrin and binds to fibronectin [30]. This is thought to facilitate adhesion of cancer cells to mesothelial cells as they secrete fibronectin [30]. Cleavage of the fibronectin into smaller fragments by cancer cell-derived metalloproteinase-2 (MMP2) facilitated this adhesion [31]. Ovarian cancer cells secrete transforming growth factor beta (TGFβ) which binds to the TGFβ receptors on the mesothelial cells, which then activate the Rac Family Small GTPase 1/SMAD (RAC1/SMAD) signaling pathway. This leads to increased expression of fibronectin and the activation of mesothelial mesenchymal transition, where mesothelial cells are transformed into mesenchymal cells secreting fibronectin and providing a better adhesive substrate for the cancer cells [31]. Blocking the interaction between α5β1-integrin and fibronectin was effective in treating ovarian cancer metastasis in a prevention as well as intervention setting. The α5β1-integrin/fibronectin interaction causes downstream activation of cMet, a receptor tyrosine kinase, independent of its ligand, hepatocyte growth factor (HGF). Activation of cMet causes increased metastasis through the activation of the focal adhesion kinase/src (FAK/Src) signaling pathway [32]. 

Since interfering with the interaction between the α5β1-integrin and fibronectin interaction was found to be effective in reducing metastasis, a clinical trial was initiated using volociximab, a humanized monoclonal antibody against the α5-integrin [33]. In 2011, Bell-McGuinn et al. published a phase II clinical trial report on its role in the treatment of platinum-resistant ovarian cancer [34]. They found that only 1 out of 13 patients had stable disease while using the drug even though it was well-tolerated upon weekly administration. Further studies are required to optimize the dosage and test if it can be effective in chemo-naive patients in combination with standard of care chemotherapy. Resveratrol, a phytoalexin produced by certain plants when damaged by a pathogen, inhibits ovarian cancer cell adhesion to mesothelial cells by decreasing α5β1-integrin levels and increasing levels of secreted hyaluronic acid [35]. Similarly, resveratrol inhibits ovarian cancer tumor growth in combination with cisplatin in mice [36]. In addition to the effects on α5β1-integrin and hyaluronic acid, resveratrol also inhibits the growth of cancer cells through apoptosis [37] and downregulation of signal transducer and activator of transcription 3 (STAT3) [38]. Extensive preclinical studies have been done with resveratrol for its potential chemo-preventive properties in colorectal cancer and hepatic metastasis [39]. Investigations are ongoing to assess its pharmacokinetics and pharmacodynamics.

### 4.2. Tissue Plasminogen Activator Inhibitor Type 1 (PAI-1) Induction

TGFβ1 from the metastasizing ovarian cancer cells can induce the mesothelial cells to secrete plasminogen activator inhibitor type 1 (PAI-1), which leads to an increased invasion of cancer cells through the mesothelial cell layer. The secretion of PAI-1 is caused by activation of mitogen-activated protein kinase (MAPK) in the mesothelial cells. The PAI-1 probably induces cancer cells invasion by promoting cell–cell interactions [40]. Neutralizing antibodies against urokinase plasminogen activator (uPA) or urokinase plasminogen activator receptor (uPAR) inhibited adhesion of the cancer cells to the mesothelial layer, while blocking PAI-1 had the opposite effect [40]. However, uPA, uPAR, and PAI-1 all increased the invasion. Interestingly, the signaling between the cancer cells and the mesothelial cells appears to be reciprocal, since interactions with the mesothelial cells also induce increased TGFβ1 in cancer cells [41]. This is through a paracrine signaling mechanism. 

Inhibition of PAI-1 leads to dissociation of PAI-1 and uPAR from α5-integrin and FAK, resulting in the inhibition of phosphorylation of FAK and extracellular signal-regulated kinase (ERK). This causes a decrease in proliferation and induction of G0/G1 cell cycle arrest, followed by apoptosis in both ovarian cancer cells. Inhibition of FAK phosphorylation also leads to inhibition of peritoneal dissemination of ovarian cancer cells [42]. Increased expression of PAI-1 is found in many ovarian cancer patient tumors and it leads to poor prognosis, supporting the importance of PAI-1 in the disease progression [43]. However, increased plasminogen expression is a favorable prognostic marker in high-grade serous ovarian cancer [44]. There is abundant evidence that uPA, its inhibitors PAI-1 and PAI-2, and its cells surface receptor, uPAR, play a fundamental role in tumor invasion and metastasis and are of significant prognostic significance for many tumor types [45]. Since many of these studies involve analysis of fully formed tumors, it reflects the importance of PAI-1, uPA and uPAR in advanced metastasis, in addition to their role in initial attachment and invasion through the mesothelium.

## 5. Mesothelial Clearance

The mesothelial cells naturally offer a non-adhesive, slippery surface. In order to establish metastatic colonization, cancer cells need to attach to and then invade through the mesothelial cell barrier in the peritoneum in order to establish metastasis [18,46]. Biopsies from advanced epithelial ovarian cancer attached to peritoneum indicate that the mesothelial cell layer in them is not continuous, and the mesothelial cells have a different morphology compared to the normal mesothelium [47]. Niedbala et al. developed an in vitro model to study the interactions between mesothelial and cancer cells [46]. Ovarian cancer cells disrupt the mesothelial cell–cell adhesions, which results in retraction of the mesothelial cells away from each other. This leads to the exposure of the extracellular matrix, to which the cancer cell can attach more efficiently [46]. Iwanicki et al. studied such mesothelial clearance using live imaging in an in vitro model [19]. Their study demonstrated that cancer cells disrupt the mesothelial cell layer using a myosin retraction force, which depended on α5-integrin and talin. Mesothelial cells in direct contact with the ovarian cancer cells migrate for longer distances than mesothelial cells that did not contact the cancer cells. Blocking α5-integrin using a neutralizing antibody decreases the mesothelial clearance, but blocking of αV- or α2-integrin, or CD44 did not have a similar effect. This suggests that cancer cell attachment to the mesothelial cell-secreted fibronectin, specifically through the α5-integrin, is essential for mesothelial cell clearance. The cancer cells attach to the mesothelial cells using the α5β1-integrin–fibronectin interaction, causing the activation of talin I, which is associated with the α5β1 integrin, and subsequently activates myosin-mediated contraction. This leads to detachment of fibronectin from the surface of the mesothelial cell layer and mesothelial clearance [19]. The same group later demonstrated that EMT correlates with the ability of the ovarian cancer cells to clear the mesothelial layer [48]. They found that cancer cell spheroids that attached to mesothelial cells contain a heterogenous population of cells with different abilities to clear the mesothelial layer. The cells with greater ability to clear the mesothelial layer exhibit high levels of mesenchymal markers. Clearance competence correlates with TGFβ-dependent induction of EMT via SMADs [48]. Exosomal miR-21 and miR-29a increase mesothelial clearance and are associated with poor survival rates [49]. Mesothelial cell clearance may also be facilitated by the migration induced in them by HGF and EGFR ligands present in malignant ascites [50]. These growth factors mediate migration of mesothelial cells via activation of cMet, which signals through MAPK and AKT to induce migration in the mesothelial cells.

## 6. Mesothelial Mesenchymal Transition (MMT)

Mesothelial cells may acquire fibroblast-like characteristics under certain conditions like inflammation or activation of repair response, in cases of peritoneal dialysis [51]. The newly formed fibroblast-like cells are able to synthesize inflammatory mediators, angiogenic factors, and altered extra cellular matrix (ECM) [52]. Cancer associated fibroblasts (CAFs) are a prominent cell population in tumors and promote most stages of cancer growth and progression. CAFs can originate by the activation of resident fibroblasts, recruitment of precursor cells derived from the bone marrow, mesenchymal stem cells or from endothelial cells [53,54,55]. However, another potential source of CAFs can be mesothelial cells undergoing MMT, similar to what happens in peritoneal dialysis patients. MMT is dependent on the TGFβ/SMAD pathway [56]. Histological observations of the peritoneal ovarian metastatic nodules identified spindle-shaped cells in the sub-mesothelial regions [54]. Immunostaining revealed that these spindle cells express both mesothelial markers and CAF markers like α-smooth muscle actin (αSMA). These results were verified using specific cytokeratin expression profiles in cancer cells and normal mesothelial cells. Culturing mesothelial cells with conditioned medium from ovarian cancer cells causes the acquisition of a spindle-like morphology, similar to mesothelial cells treated with TGFβ and IL1β. It also causes the upregulation of MMT markers like fibronectin, vascular endothelial growth factor (VEGF) and TGF β1 in mesothelial cells [54]. Malignant ascites is abundant in cytokines [56], chemokines [57], exosomes [58,59], and growth factors [50]. The presence of these factors may contribute towards increased tumor attachment, invasiveness and colonization [60,61]. In addition, they could possibly induce MMT in a manner similar to that observed in peritoneal dialysis patients [62,63]. In peritoneal dialysis patients, HGF is thought to inhibit the effect of glucose in enhancing the MMT [64]. However, in ovarian cancer patients, HGF can potentially induce the MMT [65]. 

TGFβ1 from ovarian cancer cells can also induce MMT by increasing RhoA activity. The Rho kinase (ROCK) inhibitor, Y-27632, decreases TGFβ1-induced MMT, confirming this mechanism [66,67,68]. Statins block the HMG reductase enzyme, which subsequently decrease isoprenoids, which are required by Rho GTPases [69]. Using statins decreased MMT in peritoneal dialysis patients [70]. Retrospective analysis of patients with epithelial ovarian cancer showed that using statins increased the progression free survival, after the cytoreductive therapy, by about eight months, and overall survival by about 16 months [71]. However, the improved survival may be related to inhibition of RhoA-mediated increases in invasiveness of cancer cells and not just inhibition of MMT [72]. Xie et al. conducted a meta-analysis on the effect of statins in improving the survival in patients with gynecological cancers. They did not find randomized controlled trials, however, they found 10 retrospective cohorts and one case control study. Based on these, they concluded that statins may improve the overall survival and progression free survival [73]. Astragaloside IV is a traditional Chinese herbal medication extracted from *Astragalus membranaceus*. It showed an inhibitory effect on the TGFβ1-induced MMT [74]. Astragaloside IV induces miR-134 expression, leading to a decrease in expression of cyclic adenosine monophosphate (cAMP) responsive element binding protein 1 (CREB1), which is a proto-oncogene regulating cancer progression, and promotes EMT, in colorectal cancer [75]. 

Dexamethasone inhibits TGFβ1-induced phosphorylation of MAPK and p38 MAPK. This inhibition is independent of SMADs. Interestingly, Dexamethasone reversed the TGFβ1-mediated MMT in peritoneal mesothelial cells or induced mesenchymal to epithelial transition [76]. Glucocorticoids may be used in ovarian cancer patients either to reduce nausea from the chemotherapy or to prevent the paclitaxel-induced hypersensitivity [77,78]. However, administration of glucocorticoids in ovarian cancer patients may increase the level of antiapoptotic genes, which subsequently decrease the effectiveness of chemotherapy in ovarian cancer patients [79]. Tamoxifen, a selective estrogen receptor modulator (SERM), is thought to decrease the TGFβ1-induced MMT, not reversing it, in peritoneal dialysis-derived mesothelial cells [80]. Paleari et al. conducted a meta-analysis including 53 randomized clinical trials with 2490 patients, in order to study the effect of endocrine therapy with tamoxifen and aromatase inhibitors. They found that tamoxifen showed the highest summary clinical benefit rate compared to the other therapies. The endocrine therapy carries lower risk compared to other treatments or placebo [81]. 

## 7. Metastatic Colonization

Once the metastasizing ovarian cancer cells have breached the mesothelium, they initiate the process of metastatic colonization. This involves extensive, productive cross-talk between the cancer cells and their new microenvironment [82]. Such interactions can trigger adaptive responses in the cancer cells, which enable them to adjust to the new microenvironmental factors and start establishing the metastatic tumor. Such adaptive responses include changes in gene expression, to take advantage of, or adjust to the factors present in the new microenvironment. The interactions with the omental microenvironment trigger changes in microRNAs, DNA methylation and transcription factors in the ovarian cancer cells [83,84]. The initial interactions with the mesothelial cells cause a decreased expression of the tumor suppressor microRNA, miR-193b [83]. This promotes metastatic colonization by increasing invasiveness and proliferation of the cancer cells. Overexpression of miR-193b causes a significant decrease in metastasis in mouse xenografts, while stable inhibition leads to an increase in metastasis [83]. microRNAs act through the translational inhibition of their targets. One of the key targets of miR-193b is uPA. Downregulation of miR-193b in the metastasizing ovarian cancer cells through the interactions with the mesothelium causes the concomitant increase in expression of its direct target uPA. Functional rescue experiments implicate that this increase in uPA is responsible for the increased invasiveness and proliferation of the metastasizing ovarian cancer cells [83]. Interestingly, the downregulation of miR-193b is induced by promoter hypermethylation through DNA methyl transferase 1 (DNMT1).

Transcription factors are important regulators of gene expression, and the initial interactions with the mesothelium can trigger deregulation of transcription factors, because of the signals from the new microenvironment. One of the key capabilities for successful colonization is the ability to invade into the tissue parenchyma of the metastatic organ. Several members of the erythroblast transformation-specific (ETS) family of transcription factors have the ability to increase motility of the cancer cells [85,86]. Among the oncogenic members of the ETS family, ETS 1 is the most induced in the metastasizing ovarian cancer cells [84]. This is triggered by the direct interaction with the mesothelium, which activated MAPK signaling in the cancer cells. MAPK can phosphorylate and activate ETS1, which can then transcribe itself and other targets. Among the ETS1-regulated genes in metastasizing ovarian cancer cells, protein tyrosine kinase 2 (PTK2) plays a key role in promoting metastasis [84]. PTK2 codes for FAK and activated ETS1 causes an increased expression of FAK. Interestingly, the ligand-independent activation of cMET by α5-integrin causes phosphorylation and activation of FAK during metastasis. This points toward a synergistic effect of extracellular signals for the metastatic microenvironment on both increasing the expression and activation of FAK, implicating it as a key factor in driving metastatic colonization in ovarian cancer.

## 8. Fibroblasts in Metastatic Colonization

Fibroblasts are a major cellular component and source of ECM in tumor microenvironment (TME) [87]. Depending on their site of origin and host stromal tissue type, fibroblasts display heterogeneous phenotypes by activating different transcriptional programs controlled by epigenetic modifications and paracrine signaling. It is interesting to see contradicting anti-tumorigenic function of normal fibroblast and pro-tumorigenic responses of CAFs [88,89,90]. Normal fibroblasts play an essential role in organizing ECM by altering expression and activities of adhesion molecules, other cellular components and fine-tuning the balance of various proteolytic enzymes and tissue inhibitors. Maintaining normal stromal architecture and configuring normal epithelial phenotype by fibroblasts is an important defense against metastatic colonization. Normal fibroblasts also cause “neighbor suppression” by inhibiting the growth of adjacent abnormal or transformed cells [91]. Contact-independent tumor suppression is accomplished via different cytokines and chemokines such as tumor necrosis factor-alpha (TNF-alpha), IL-6, and TGF-beta released by fibroblasts. Fibroblasts are also a key constituent of the basement membrane of the omentum and the peritoneum [82]. They secret the ECM that form the basement membrane, and multiple other factors that are trapped in the ECM. These fibroblasts are among the first cells encountered by the metastasizing ovarian cancer cells. Interactions with such resident normal fibroblasts can induce changes in microRNAs in the ovarian cancer cells that can help them adapt to the new microenvironment (unpublished data). At the same time, the cancer cells have the ability to reprogram these normal fibroblasts into CAF by altering the expression of a set of microRNAs in them [92]. The cancer cell-induced decrease in the expression of miR-214 and miR-31 and an increase in the expression of mi-R155 converts normal fibroblasts into CAFs. Interestingly, CAFs could be converted back into normal fibroblasts by the combined over-expression of miR-214 and miR-31 and the inhibition of miR-155. The CAFs then promote cancer cells invasion and proliferation by secreting several chemokines and cytokines including C-C motif chemokine ligand 5 (CCL5), which is a direct target of miR-214. Inhibition of CCL5 in nude mice co-injected with ovarian cancer cells and CAFs significantly decreased the ability of the CAFs to promote tumor growth and metastasis [92]. Recent proteomics studies have revealed that the methyltransferase nicotinamide N-methyltransferase can also help reprogram CAFs into an activated state that supports metastasis [93].

The ability to reprogram the resident normal fibroblasts is key to successfully establishing the metastasis. Once activated, the CAFs secrete many factors that remodel the ECM, promote cancer cell growth and invasion, trigger angiogenesis, and have immunomodulatory functions, together promoting metastasis [87]. Other cell types, such as fibrocytes, epithelial cells (through EMT), mesenchymal stem cells, pericytes, adipocytes, and endothelial progenitor cells can differentiate and provide the source of CAFs as well [94]. This switch of normal stroma into CAF-containing TME is one of the fundamental steps promoting tumor development. In addition to secreting growth factors including hepatocyte growth factor (HGF), stromal-derived factor-1 (SDF-1) and fibroblast growth factor (FGF), that directly affect cell motility, CAFs are the source of ECM-degrading proteases such as the MMPs. Cancer cell invasion through the basement membrane is a complex process, involving stepwise adhesion and proteolysis. However, basement membrane invasion can also be achieved by CAFs pulling and stretching their plasma membranes [95,96]. This creates gaps in the basement membrane that allow the cancer cells to invade even without proteolytic MMP activity [95]. CAFs also facilitate stromal dissemination of cancer cells that have not completely undergone EMT, by paving the way and forming ECM tracks [97,98]. The CAF-directed cancer invasion through stroma can utilize matrix-degrading proteases at the surface of the leader fibroblasts. At the metastatic site, depletion of all four members of tissue inhibitor of metalloproteinases (TIMP) causes activation of CAFs and secretion of exosomes containing MMPs, miR-45 and other ECM proteins. Paracrine signaling via chemokines such as IL-6, IL-11 produced by CAFs activates the janus kinase2/signal transducer and activator of transcription 3 (JAK2/STAT3) pathway, which enhances the ability of cancer cells to undergo EMT and facilitates growth and metastasis to peritoneum [99,100]. C-X-C motif chemokine 12 (CXCL12) secreted by CAFs can also induce EMT in oral squamous cell carcinoma and breast cancer models [101,102]. 

While this review focusses on metastatic colonization, the CAFs can also play a role in the dissemination of cancer cells from the primary tumor to the site of peritoneal metastasis. The exfoliated ovarian cancer cells floating in the ascites as spheroids contain CAFs as well as activated mesothelial cells, which contribute to the development of ascitic microenvironment, assist cancer cell survival and subsequent colonization of the metastatic site. These spheroids represent the invasive and chemoresistant cellular population. They also contain various acellular elements like cytokines and ECMs that contribute to the development of the ascitic microenvironment, assist cancer cell survival and subsequent colonization of the metastatic site [9,103,104]. Ascitic tumor cells in HGSOC are characterized by high levels of α_5_-integrin expression, which tend to form heterotypic spheroids with fibroblasts. There is a reciprocal cooperation between the cancer cells and fibroblasts, where ovarian cancer cells with high α_5_-integrin expression are selectively recruited by CAFs to form the unique heterotypic spheroids. Similarly, activated CAFs secrete EGF that supports survival of the cancer cells, direct their peritoneal and transperitoneal adhesion and invasion and finally, constitute the tumor stroma in newly formed metastases [9]. 

Cancer cells can prime and recruit stromal components such as fibroblasts at the distant metastatic site (the PMN) to secrete ECM proteins such as fibronectin. Fibronectin, an extracellular matrix glycoprotein involved in numerous cellular processes including embryonic cell migration and vascular development, also appears to be an important component of PMN. In pancreatic ductal adenocarcinoma (PDAC) metastasizing to liver, resident hepatic stellate cells can be activated into periostin-secreting myofibroblasts through granulin secreted by tumor-associated macrophages (TAMs) [105]. At the metastatic niche, fibroblast-specific protein 1 (FSP1)-positive cells have also been found to enhance cancer cell metastasis via vascular endothelial growth factor A (VEGFA) secretion, and depletion of these cells significantly reduces the metastatic colonization, while primary tumor growth remains unaffected [106]. CAFs also promote neoangiogenesis in metastatic TME by modulating connective tissue growth and micro-vessel density [91]. CAFs in the omental metastasis can also play an important role in the metabolic switch happening in the cancer cells, as a result of depletion of available fatty acids, as the tumor progresses [107]. CAFs caused the activation of phosphoglucomutase 1 in the cancer cells, enabling them to utilize glycogen as an energy source instead. Overall, CAFs appear to be important contributors towards the initial steps of ovarian cancer metastatic colonization, as well as in advanced metastasis.

## 9. Role of Adipocytes in Metastatic Colonization

An important and abundant cellular component of the omentum is the adipocyte. Omental adipocytes secrete adipokines such as IL-8, IL-6, monocyte chemoattractant protein-1 and adiponectin, which promote homing of metastasizing ovarian cancer cells to omentum [108]. Cancer cells affect metabolism in adipocytes by inducing hydrolysis of triglycerides, stored in adipocyte lipid droplets, into free fatty acids and glycerol [108]. The glycerol can potentially be fed into the glycolytic pathway and facilitate metabolic adaptation of the tumor in the new metastatic niche. The adipocytes also induce the expression of FABP4, a fatty acid transporter, in the cancer cells. Interestingly, FABP4 is also present in endothelial cells, where it appears to regulate cell proliferation and angiogenesis. The lipid transfer from adipocytes induces β-oxidation in tumor cells and enhances their proliferation in vitro [108]. The adipocytes also induce the expression of CD36 in the ovarian cancer cells that help uptake of the fatty acids and the eventual utilization to promote metastatic growth [109]. In addition, adipocytes induce calcium-dependent activation salt-inducible kinase 2 in the ovarian cancer cells, activating the phosphoinositide 3-kinase/AKT (PI3K/AKT) pathway and phosphorylating acetyl-CoA carboxylase, promoting metastasis [110]. Essentially, it appears that cancer cells force adipocytes to ‘give up’ their lipids. Through paracrine secretions and the abundance of readily transferable lipids, adipocytes create a microenvironment that alters tumor metabolism and supports phenotypes that are more aggressive. This process eventually leads to the transformation of omentum from a fat pad into a solid tumor mass with few remaining adipocytes, a process often referred to as ‘omental caking’. Interestingly, targeting the adipocytes with a DNA methyltransferase (DNMT) inhibitor can affect their ability to promote cancer cell migration and proliferation [111].

Adipocytes also interact with peritoneal macrophages and facilitate their activation to a tumor-associated phenotype (tumor-associated macrophage). It has been demonstrated that adipocyte-supplied poly-unsaturated fatty acids (PUFAs) serve as ligands for transcription factor peroxisome proliferator-activated receptor β/δ (PPARβ/δ) in macrophages [112]. This fatty acid accumulation leads to transcriptional deregulation and pro-tumorigenic phenotype in peritoneal macrophages. These macrophages contribute to the formation of new “milky spots” around the homed tumor cells in omentum [113,114]. Once adhered, the tumor cells deplete the adipocytes and induce hypoxia, which leads to increased angiogenesis, and advances metastatic progression even when fat stores are depleted [115]. Omental fat cells also activate pro-survival pathways, p38 and STAT3, in the ovarian cancer cells, indicating the potential importance of an adipocyte-rich environment for homing and growth of tumor cells in the omentum.

Secrete adipokines are shown in Table 1 together with other secreted factors.

## 10. Conclusions

**Figure 2:**
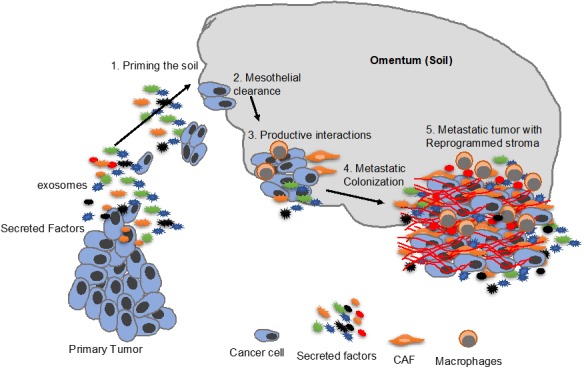
An overview of ovarian cancer metastatic colonization. Starting with priming of the metastatic niche by secreted factors, including exosomes, from the primary tumor (1), followed by mesothelial clearance (2) and reciprocal interactions between cancer cells and the microenvironment. This eventually results in metastatic colonization (4) and reprogramming of the stroma into an ‘activated’ tumor-promoting stroma (5).

In conclusion, the process of metastasis involves productive interactions with both the cellular and acellular microenvironmental components. The use of effective three-dimensional (3D) culture models in the recent past have improved our understanding of the mechanisms of such interactions and the roles that they play in metastasis. The reciprocal interactions between the metastasizing ovarian cancer cells and the microenvironment of the metastatic site may be initiated very early in the form of secreted factors produced by the primary tumor to prime the microenvironment to develop into a PMN that produces factors that induce homing of the metastasizing cells and the subsequent establishment of the disseminated tumor (Figure 2). At the metastatic site, the cross-talk can be mediated through paracrine and juxtacrine signaling that helps establish the tumor and reprogram the microenvironment into an ‘activated’ tumor promoting stroma. This activated stroma continues to play an important role in the progression of metastasis. However, much remains to be learned and further studies are needed to fully understand the reciprocal interactions that are essential for successful metastasis. As our knowledge in this area increases, it will help identify novel therapeutic targets that are potentially effective in treating ovarian cancer metastasis. Since a majority of ovarian cancer patients already have metastatic disease at the time of diagnosis, such novel therapies may help improve their outcome.

## Figures and Tables

**Table 1:** Secreted factors involved in reciprocal interactions between metastasizing ovarian cancer cells and the microenvironment.

S.No	Secreted Factors	Sources	References
1	Transforming growth factor-β (TGF-β)	Mesothelial cells	[12,16]
2	Transforming growth factor-β (TGF-β)	Cancer cells	[71]
3	Platelet-derived growth factor (PDGF)	Mesothelial cells	[12,16]
4	Fibroblast growth factor (FGF)	Mesothelial cells	[12,16]
5	Hepatocyte growth factor (HGF)	Mesothelial cells	[12,16]
6	Keratinocyte growth factor (KGF)	Mesothelial cells	[12,16]
7	Epithelial growth factors (EGFs)	Mesothelial cells	[12,16]
8	Cytokines like IL6, stroma derived factor 1 (SDF1), leucine leucine 37 (LL 37)	Cancer cells	[27,28]
9	Fibronectin	Mesothelial cells	[71]
10	Matrix metalloprotease 9 (MMP 9)	Fibroblasts	[30,97]
11	Plasminogen activator inhibitor type 1(PAI-1)	Mesothelial cells	[43]
12	Urokinase plasminogen activator (uPA)		[53]
13	Cytokines (angiogenin, angiopoietin-2, GRO, ICAM-1, IL-6, IL-6R, IL-8, IL-10, leptin, MCP-1, MIF NAP-2, osteprotegerin (OPG), RANTES, TIMP-2 and UPAR were elevated in most malignant ascites)	Malignant ascites	[53]
14	Chemokines (CCL2, -3, -4, -5, -8, and -22)	Malignant ascites	[60]
15	Chemokines (IL-6, IL-11)	Cancer associated fibroblasts	[101]
16	Exosomes/Membrane vesicles/microvesicles	Malignant ascites	[61,62]
17	Fatty acid binding protein 4 (FABP4)	Adipocytes	[110]
18	Adipokines (IL-8, IL-6), monocyte chemoattractant protein-1 and adiponectin	Adipocytes	[110]

## References

[1] Siegel R.L., Miller K.D., Jemal A. (2019). Cancer statistics, 2019. CA Cancer J. Clin..

[2] Torre L.A., Trabert B., DeSantis C.E. (2018). Ovarian cancer statistics, 2018. CA Cancer J. Clin..

[3] Grossman D.C., Curry S.J., Owens D.K., Barry M.J., Davidson K.W., Doubeni C.A., Epling J.W., Kemper A.R., Krist A.H., Kurth A.E. (2018). Screening for Ovarian Cancer: US Preventive Services Task Force Recommendation Statement. JAMA.

[4] Kaku T., Ogawa S., Kawano Y., Ohishi Y., Kobayashi H., Hirakawa T., Nakano H. (2003). Histological classification of ovarian cancer. Med. Electron. Microsc..

[5] Prat J. (2014). Staging classification for cancer of the ovary, fallopian tube, and peritoneum. Int. J. Gynaecol. Obstet..

[6] Visvanathan K., Shaw P., May B.J., Bahadirli-Talbott A., Kaushiva A., Risch H., Narod S., Wang T.L., Parkash V., Vang R. (2018). Fallopian Tube Lesions in Women at High Risk for Ovarian Cancer: A Multicenter Study. Cancer Prev. Res..

[7] Eckert M.A., Pan S., Hernandez K.M., Loth R.M., Andrade J., Volchenboum S.L., Faber P., Montag A., Lastra R., Peter M.E. (2016). Genomics of Ovarian Cancer Progression Reveals Diverse Metastatic Trajectories Including Intraepithelial Metastasis to the Fallopian Tube. Cancer Discov..

[8] Tan D.S., Agarwal R., Kaye S.B. (2006). Mechanisms of transcoelomic metastasis in ovarian cancer. Lancet Oncol..

[9] Gao Q., Yang Z., Xu S., Li X., Yang X., Jin P., Liu Y., Zhou X., Zhang T., Gong C. (2019). Heterotypic CAF-tumor spheroids promote early peritoneal metastatis of ovarian cancer. J. Exp. Med..

[10] Pradeep S., Kim S.W., Wu S.Y., Nishimura M., Chaluvally-Raghavan P., Miyake T., Pecot C.V., Kim S.J., Choi H.J., Bischoff F.Z. (2014). Hematogenous metastasis of ovarian cancer: Rethinking mode of spread. Cancer Cell.

[11] Blackburn S.C., Stanton M.P. (2014). Anatomy and physiology of the peritoneum. Semin. Pediatr. Surg..

[12] Mutsaers S.E., Prele C.M., Lansley S.M., Herrick S.E. (2007). The origin of regenerating mesothelium: A historical perspective. Int. J. Artif. Organs.

[13] Mutsaers S.E., Wilkosz S. (2007). Structure and function of mesothelial cells. Cancer Treat. Res..

[14] Mutsaers S.E., Prele C.M., Pengelly S., Herrick S.E. (2016). Mesothelial cells and peritoneal homeostasis. Fertil. Steril..

[15] Jonjic N., Peri G., Bernasconi S., Sciacca F.L., Colotta F., Pelicci P., Lanfrancone L., Mantovani A. (1992). Expression of adhesion molecules and chemotactic cytokines in cultured human mesothelial cells. J. Exp. Med..

[16] Warn R., Harvey P., Warn A., Foley-Comer A., Heldin P., Versnel M., Arakaki N., Daikuhara Y., Laurent G.J., Herrick S.E. (2001). HGF/SF induces mesothelial cell migration and proliferation by autocrine and paracrine pathways. Exp. Cell Res..

[17] Foley-Comer A.J., Herrick S.E., Al-Mishlab T., Prele C.M., Laurent G.J., Mutsaers S.E. (2002). Evidence for incorporation of free-floating mesothelial cells as a mechanism of serosal healing. J. Cell Sci..

[18] Kenny H.A., Nieman K.M., Mitra A.K., Lengyel E. (2011). The first line of intra-abdominal metastatic attack: Breaching the mesothelial cell layer. Cancer Discov..

[19] Iwanicki M.P., Davidowitz R.A., Ng M.R., Besser A., Muranen T., Merritt M., Danuser G., Ince T.A., Brugge J.S. (2011). Ovarian cancer spheroids use myosin-generated force to clear the mesothelium. Cancer Discov..

[20] Paget S. (1989). The distribution of secondary growths in cancer of the breast. 1889. Cancer Metastasis Rev..

[21] Langley R.R., Fidler I.J. (2011). The seed and soil hypothesis revisited—The role of tumor-stroma interactions in metastasis to different organs. Int. J. Cancer.

[22] Hart I.R., Talmadge J.E., Fidler I.J. (1981). Metastatic behavior of a murine reticulum cell sarcoma exhibiting organ-specific growth. Cancer Res..

[23] Coffman L.G., Burgos-Ojeda D., Wu R., Cho K., Bai S., Buckanovich R.J. (2016). New models of hematogenous ovarian cancer metastasis demonstrate preferential spread to the ovary and a requirement for the ovary for abdominal dissemination. Transl. Res..

[24] Fong M.Y., Zhou W., Liu L., Alontaga A.Y., Chandra M., Ashby J., Chow A., O’Connor S.T., Li S., Chin A.R. (2015). Breast-cancer-secreted miR-122 reprograms glucose metabolism in premetastatic niche to promote metastasis. Nat. Cell Biol..

[25] Hoshino A., Costa-Silva B., Shen T.L., Rodrigues G., Hashimoto A., Tesic Mark M., Molina H., Kohsaka S., Di Giannatale A., Ceder S. (2015). Tumour exosome integrins determine organotropic metastasis. Nature.

[26] Peinado H., Zhang H., Matei I.R., Costa-Silva B., Hoshino A., Rodrigues G., Psaila B., Kaplan R.N., Bromberg J.F., Kang Y. (2017). Pre-metastatic niches: Organ-specific homes for metastases. Nat. Rev. Cancer.

[27] Coffelt S.B., Marini F.C., Watson K., Zwezdaryk K.J., Dembinski J.L., LaMarca H.L., Tomchuck S.L., Honer zu Bentrup K., Danka E.S., Henkle S.L. (2009). The pro-inflammatory peptide LL-37 promotes ovarian tumor progression through recruitment of multipotent mesenchymal stromal cells. Proc. Natl. Acad. Sci. USA.

[28] Chow M.T., Luster A.D. (2014). Chemokines in cancer. Cancer Immunol. Res..

[29] Yasumoto K., Koizumi K., Kawashima A., Saitoh Y., Arita Y., Shinohara K., Minami T., Nakayama T., Sakurai H., Takahashi Y. (2006). Role of the CXCL12/CXCR4 axis in peritoneal carcinomatosis of gastric cancer. Cancer Res..

[30] Sawada K., Mitra A.K., Radjabi A.R., Bhaskar V., Kistner E.O., Tretiakova M., Jagadeeswaran S., Montag A., Becker A., Kenny H.A. (2008). Loss of E-cadherin promotes ovarian cancer metastasis via alpha 5-integrin, which is a therapeutic target. Cancer Res..

[31] Kenny H.A., Kaur S., Coussens L.M., Lengyel E. (2008). The initial steps of ovarian cancer cell metastasis are mediated by MMP-2 cleavage of vitronectin and fibronectin. J. Clin. Investig..

[32] Mitra A.K., Sawada K., Tiwari P., Mui K., Gwin K., Lengyel E. (2011). Ligand-independent activation of c-Met by fibronectin and alpha(5)beta(1)-integrin regulates ovarian cancer invasion and metastasis. Oncogene.

[33] Almokadem S., Belani C.P. (2012). Volociximab in Cancer. Expert Opin. Biol. Ther..

[34] Bell-McGuinn K.M., Matthews C.M., Ho S.N., Barve M., Gilbert L., Penson R.T., Lengyel E., Palaparthy R., Gilder K., Vassos A. (2011). A phase II, single-arm study of the anti-alpha5beta1 integrin antibody volociximab as monotherapy in patients with platinum-resistant advanced epithelial ovarian or primary peritoneal cancer. Gynecol. Oncol..

[35] Mikula-Pietrasik J., Sosinska P., Ksiazek K. (2014). Resveratrol inhibits ovarian cancer cell adhesion to peritoneal mesothelium in vitro by modulating the production of alpha5beta1 integrins and hyaluronic acid. Gynecol. Oncol..

[36] Tan L., Wang W., He G., Kuick R.D., Gossner G., Kueck A.S., Wahl H., Opipari A.W., Liu J.R. (2016). Resveratrol inhibits ovarian tumor growth in an in vivo mouse model. Cancer.

[37] Lang F., Qin Z., Li F., Zhang H., Fang Z., Hao E. (2015). Apoptotic Cell Death Induced by Resveratrol Is Partially Mediated by the Autophagy Pathway in Human Ovarian Cancer Cells. PLoS ONE.

[38] Zhong L.X., Li H., Wu M.L., Liu X.Y., Zhong M.J., Chen X.Y., Liu J., Zhang Y. (2015). Inhibition of STAT3 signaling as critical molecular event in resveratrol-suppressed ovarian cancer cells. J. Ovarian Res..

[39] Howells L.M., Berry D.P., Elliott P.J., Jacobson E.W., Hoffmann E., Hegarty B., Brown K., Steward W.P., Gescher A.J. (2011). Phase I randomized, double-blind pilot study of micronized resveratrol (SRT501) in patients with hepatic metastases—Safety, pharmacokinetics, and pharmacodynamics. Cancer Prev. Res..

[40] Hirashima Y., Kobayashi H., Suzuki M., Tanaka Y., Kanayama N., Terao T. (2003). Transforming growth factor-beta1 produced by ovarian cancer cell line HRA stimulates attachment and invasion through an up-regulation of plasminogen activator inhibitor type-1 in human peritoneal mesothelial cells. J. Biol. Chem..

[41] Dasari S., Pandhiri T., Haley J., Lenz D., Mitra A.K. (2018). A Proximal Culture Method to Study Paracrine Signaling Between Cells. JoVE.

[42] Ward K.K., Tancioni I., Lawson C., Miller N.L., Jean C., Chen X.L., Uryu S., Kim J., Tarin D., Stupack D.G. (2013). Inhibition of focal adhesion kinase (FAK) activity prevents anchorage-independent ovarian carcinoma cell growth and tumor progression. Clin. Exp. Metastasis.

[43] Nakatsuka E., Sawada K., Nakamura K., Yoshimura A., Kinose Y., Kodama M., Hashimoto K., Mabuchi S., Makino H., Morii E. (2017). Plasminogen activator inhibitor-1 is an independent prognostic factor of ovarian cancer and IMD-4482, a novel plasminogen activator inhibitor-1 inhibitor, inhibits ovarian cancer peritoneal dissemination. Oncotarget.

[44] Zhao S., Dorn J., Napieralski R., Walch A., Diersch S., Kotzsch M., Ahmed N., Hooper J.D., Kiechle M., Schmitt M. (2017). Plasmin(ogen) serves as a favorable biomarker for prediction of survival in advanced high-grade serous ovarian cancer. Biol. Chem..

[45] Van Dam P.A., Coelho A., Rolfo C. (2017). Is there a role for urokinase-type plasminogen activator inhibitors as maintenance therapy in patients with ovarian cancer?. Eur. J. Surg. Oncol..

[46] Niedbala M.J., Crickard K., Bernacki R.J. (1985). Interactions of human ovarian tumor cells with human mesothelial cells grown on extracellular matrix. An in vitro model system for studying tumor cell adhesion and invasion. Exp. Cell Res..

[47] Zhang X.Y., Pettengell R., Nasiri N., Kalia V., Dalgleish A.G., Barton D.P. (1999). Characteristics and growth patterns of human peritoneal mesothelial cells: Comparison between advanced epithelial ovarian cancer and non-ovarian cancer sources. J. Soc. Gynecol. Investig..

[48] Davidowitz R.A., Selfors L.M., Iwanicki M.P., Elias K.M., Karst A., Piao H., Ince T.A., Drage M.G., Dering J., Konecny G.E. (2014). Mesenchymal gene program-expressing ovarian cancer spheroids exhibit enhanced mesothelial clearance. J. Clin. Investig..

[49] Vaksman O., Trope C., Davidson B., Reich R. (2014). Exosome-derived miRNAs and ovarian carcinoma progression. Carcinogenesis.

[50] Matte I., Lane D., Laplante C., Garde-Granger P., Rancourt C., Piche A. (2015). Ovarian cancer ascites enhance the migration of patient-derived peritoneal mesothelial cells via cMet pathway through HGF-dependent and -independent mechanisms. Int. J. Cancer.

[51] Lee Y.C., Tsai Y.S., Hung S.Y., Lin T.M., Lin S.H., Liou H.H., Liu H.C., Chang M.Y., Wang H.H., Ho L.C. (2014). Shorter daily dwelling time in peritoneal dialysis attenuates the epithelial-to-mesenchymal transition of mesothelial cells. BMC Nephrol..

[52] Yanez-Mo M., Lara-Pezzi E., Selgas R., Ramirez-Huesca M., Dominguez-Jimenez C., Jimenez-Heffernan J.A., Aguilera A., Sanchez-Tomero J.A., Bajo M.A., Alvarez V. (2003). Peritoneal dialysis and epithelial-to-mesenchymal transition of mesothelial cells. N. Engl. J. Med..

[53] Kalluri R., Zeisberg M. (2006). Fibroblasts in cancer. Nat. Rev. Cancer.

[54] Sandoval P., Jimenez-Heffernan J.A., Rynne-Vidal A., Perez-Lozano M.L., Gilsanz A., Ruiz-Carpio V., Reyes R., Garcia-Bordas J., Stamatakis K., Dotor J. (2013). Carcinoma-associated fibroblasts derive from mesothelial cells via mesothelial-to-mesenchymal transition in peritoneal metastasis. J. Pathol..

[55] Potenta S., Zeisberg E., Kalluri R. (2008). The role of endothelial-to-mesenchymal transition in cancer progression. Br. J. Cancer.

[56] Matte I., Lane D., Laplante C., Rancourt C., Piche A. (2012). Profiling of cytokines in human epithelial ovarian cancer ascites. Am. J. Cancer Res..

[57] Milliken D., Scotton C., Raju S., Balkwill F., Wilson J. (2002). Analysis of chemokines and chemokine receptor expression in ovarian cancer ascites. Clin. Cancer Res..

[58] Graves L.E., Ariztia E.V., Navari J.R., Matzel H.J., Stack M.S., Fishman D.A. (2004). Proinvasive properties of ovarian cancer ascites-derived membrane vesicles. Cancer Res..

[59] Choi D.S., Park J.O., Jang S.C., Yoon Y.J., Jung J.W., Choi D.Y., Kim J.W., Kang J.S., Park J., Hwang D. (2011). Proteomic analysis of microvesicles derived from human colorectal cancer ascites. Proteomics.

[60] Freedman R.S., Deavers M., Liu J., Wang E. (2004). Peritoneal inflammation—A microenvironment for Epithelial Ovarian Cancer (EOC). J. Transl. Med..

[61] Matte I., Lane D., Bachvarov D., Rancourt C., Piche A. (2014). Role of malignant ascites on human mesothelial cells and their gene expression profiles. BMC Cancer.

[62] Demir A.Y., Groothuis P.G., Dunselman G.A., Schurgers L., Evers J.L., de Goeij A.F. (2005). Molecular characterization of soluble factors from human menstrual effluent that induce epithelial to mesenchymal transitions in mesothelial cells. Cell Tissue Res..

[63] Lopez-Cabrera M. (2014). Mesenchymal Conversion of Mesothelial Cells Is a Key Event in the Pathophysiology of the Peritoneum during Peritoneal Dialysis. Adv. Med..

[64] Yu M.A., Shin K.S., Kim J.H., Kim Y.I., Chung S.S., Park S.H., Kim Y.L., Kang D.H. (2009). HGF and BMP-7 ameliorate high glucose-induced epithelial-to-mesenchymal transition of peritoneal mesothelium. J. Am. Soc. Nephrol..

[65] Nakamura M., Ono Y.J., Kanemura M., Tanaka T., Hayashi M., Terai Y., Ohmichi M. (2015). Hepatocyte growth factor secreted by ovarian cancer cells stimulates peritoneal implantation via the mesothelial-mesenchymal transition of the peritoneum. Gynecol. Oncol..

[66] Manickam N., Patel M., Griendling K.K., Gorin Y., Barnes J.L. (2014). RhoA/Rho kinase mediates TGF-beta1-induced kidney myofibroblast activation through Poldip2/Nox4-derived reactive oxygen species. Am. J. Physiol. Ren. Physiol..

[67] Zhang H., Liu X., Liu Y., Yi B., Yu X. (2011). Epithelial-mesenchymal transition of rat peritoneal mesothelial cells via Rhoa/Rock pathway. In Vitro Cell. Dev. Biol. Anim..

[68] Kenny H.A., Chiang C.Y., White E.A., Schryver E.M., Habis M., Romero I.L., Ladanyi A., Penicka C.V., George J., Matlin K. (2014). Mesothelial cells promote early ovarian cancer metastasis through fibronectin secretion. J. Clin. Investig..

[69] Rikitake Y., Liao J.K. (2005). Rho GTPases, statins, and nitric oxide. Circ. Res..

[70] Chang T.I., Kang H.Y., Kim K.S., Lee S.H., Nam B.Y., Paeng J., Kim S., Park J.T., Yoo T.H., Kang S.W. (2014). The effect of statin on epithelial-mesenchymal transition in peritoneal mesothelial cells. PLoS ONE.

[71] Elmore R.G., Ioffe Y., Scoles D.R., Karlan B.Y., Li A.J. (2008). Impact of statin therapy on survival in epithelial ovarian cancer. Gynecol. Oncol..

[72] Horiuchi A., Kikuchi N., Osada R., Wang C., Hayashi A., Nikaido T., Konishi I. (2008). Overexpression of RhoA enhances peritoneal dissemination: RhoA suppression with Lovastatin may be useful for ovarian cancer. Cancer Sci..

[73] Xie W., Ning L., Huang Y., Liu Y., Zhang W., Hu Y., Lang J., Yang J. (2017). Statin use and survival outcomes in endocrine-related gynecologic cancers: A systematic review and meta-analysis. Oncotarget.

[74] Zhang L., Li Z., He W., Xu L., Wang J., Shi J., Sheng M. (2015). Effects of Astragaloside IV Against the TGF-beta1-Induced Epithelial-to-Mesenchymal Transition in Peritoneal Mesothelial Cells by Promoting Smad 7 Expression. Cell. Physiol. Biochem..

[75] Ye Q., Su L., Chen D., Zheng W., Liu Y. (2017). Astragaloside IV Induced miR-134 Expression Reduces EMT and Increases Chemotherapeutic Sensitivity by Suppressing CREB1 Signaling in Colorectal Cancer Cell Line SW-480. Cell. Physiol. Biochem..

[76] Jang Y.H., Shin H.S., Sun Choi H., Ryu E.S., Jin Kim M., Ki Min S., Lee J.H., Kook Lee H., Kim K.H., Kang D.H. (2013). Effects of dexamethasone on the TGF-beta1-induced epithelial-to-mesenchymal transition in human peritoneal mesothelial cells. Lab. Investig..

[77] Yanaranop M., Chaithongwongwatthana S. (2016). Intravenous versus oral dexamethasone for prophylaxis of paclitaxel-associated hypersensitivity reaction in patients with primary ovarian, fallopian tube and peritoneal cancer: A double-blind randomized controlled trial. Asia Pac. J. Clin. Oncol..

[78] Sorbe B., Hallen C. (1991). Betamethasone-dixyrazine versus betamethasone-metoclopramide as antiemetic treatment of cisplatin-doxorubicin-induced nausea in ovarian carcinoma patients. Eur. J. Gynaecol. Oncol..

[79] Melhem A., Yamada S.D., Fleming G.F., Delgado B., Brickley D.R., Wu W., Kocherginsky M., Conzen S.D. (2009). Administration of glucocorticoids to ovarian cancer patients is associated with expression of the anti-apoptotic genes SGK1 and MKP1/DUSP1 in ovarian tissues. Clin. Cancer Res..

[80] Loureiro J., Sandoval P., del Peso G., Gonzalez-Mateo G., Fernandez-Millara V., Santamaria B., Bajo M.A., Sanchez-Tomero J.A., Guerra-Azcona G., Selgas R. (2013). Tamoxifen ameliorates peritoneal membrane damage by blocking mesothelial to mesenchymal transition in peritoneal dialysis. PLoS ONE.

[81] Paleari L., Gandini S., Provinciali N., Puntoni M., Colombo N., DeCensi A. (2017). Clinical benefit and risk of death with endocrine therapy in ovarian cancer: A comprehensive review and meta-analysis. Gynecol. Oncol..

[82] Mitra A.K. (2016). Ovarian Cancer Metastasis: A Unique Mechanism of Dissemination. Tumor Metastasis.

[83] Mitra A.K., Chiang C.Y., Tiwari P., Tomar S., Watters K.M., Peter M.E., Lengyel E. (2015). Microenvironment-induced downregulation of miR-193b drives ovarian cancer metastasis. Oncogene.

[84] Tomar S., Plotnik J.P., Haley J., Scantland J., Dasari S., Sheikh Z., Emerson R., Lenz D., Hollenhorst P.C., Mitra A.K. (2018). ETS1 induction by the microenvironment promotes ovarian cancer metastasis through focal adhesion kinase. Cancer Lett..

[85] Kar A., Gutierrez-Hartmann A. (2013). Molecular mechanisms of ETS transcription factor-mediated tumorigenesis. Crit. Rev. Biochem. Mol. Biol..

[86] Seth A., Watson D.K. (2005). ETS transcription factors and their emerging roles in human cancer. Eur. J. Cancer.

[87] Dasari S., Fang Y., Mitra A.K. (2018). Cancer Associated Fibroblasts: Naughty Neighbors That Drive Ovarian Cancer Progression. Cancers.

[88] Gascard P., Tlsty T.D. (2016). Carcinoma-associated fibroblasts: Orchestrating the composition of malignancy. Genes Dev..

[89] Santi A., Kugeratski F.G., Zanivan S. (2018). Cancer Associated Fibroblasts: The Architects of Stroma Remodeling. Proteomics.

[90] Chen X., Song E. (2019). Turning foes to friends: Targeting cancer-associated fibroblasts. Nat. Rev. Drug Discov..

[91] Alkasalias T., Moyano-Galceran L., Arsenian-Henriksson M., Lehti K. (2018). Fibroblasts in the Tumor Microenvironment: Shield or Spear?. Int. J. Mol. Sci..

[92] Mitra A.K., Zillhardt M., Hua Y., Tiwari P., Murmann A.E., Peter M.E., Lengyel E. (2012). MicroRNAs reprogram normal fibroblasts into cancer-associated fibroblasts in ovarian cancer. Cancer Discov..

[93] Eckert M.A., Coscia F., Chryplewicz A., Chang J.W., Hernandez K.M., Pan S., Tienda S.M., Nahotko D.A., Li G., Blazenovic I. (2019). Proteomics reveals NNMT as a master metabolic regulator of cancer-associated fibroblasts. Nature.

[94] Orimo A., Weinberg R.A. (2006). Stromal fibroblasts in cancer: A novel tumor-promoting cell type. Cell Cycle.

[95] Glentis A., Oertle P., Mariani P., Chikina A., El Marjou F., Attieh Y., Zaccarini F., Lae M., Loew D., Dingli F. (2017). Cancer-associated fibroblasts induce metalloprotease-independent cancer cell invasion of the basement membrane. Nat. Commun..

[96] Sherwood D.R., Plastino J. (2018). Invading, Leading and Navigating Cells in Caenorhabditis elegans: Insights into Cell Movement in Vivo. Genetics.

[97] Friedl P., Locker J., Sahai E., Segall J.E. (2012). Classifying collective cancer cell invasion. Nat. Cell Biol..

[98] Mayor R., Etienne-Manneville S. (2016). The front and rear of collective cell migration. Nat. Rev. Mol. Cell Biol..

[99] Ollila S., Domenech-Moreno E., Laajanen K., Wong I.P., Tripathi S., Pentinmikko N., Gao Y., Yan Y., Niemela E.H., Wang T.C. (2018). Stromal Lkb1 deficiency leads to gastrointestinal tumorigenesis involving the IL-11-JAK/STAT3 pathway. J. Clin. Investig..

[100] Wu X., Tao P., Zhou Q., Li J., Yu Z., Wang X., Li J., Li C., Yan M., Zhu Z. (2017). IL-6 secreted by cancer-associated fibroblasts promotes epithelial-mesenchymal transition and metastasis of gastric cancer via JAK2/STAT3 signaling pathway. Oncotarget.

[101] Onoue T., Uchida D., Begum N.M., Tomizuka Y., Yoshida H., Sato M. (2006). Epithelial-mesenchymal transition induced by the stromal cell-derived factor-1/CXCR4 system in oral squamous cell carcinoma cells. Int. J. Oncol..

[102] Soon P.S., Kim E., Pon C.K., Gill A.J., Moore K., Spillane A.J., Benn D.E., Baxter R.C. (2013). Breast cancer-associated fibroblasts induce epithelial-to-mesenchymal transition in breast cancer cells. Endocr. Relat. Cancer.

[103] Ahmed N., Stenvers K.L. (2013). Getting to know ovarian cancer ascites: Opportunities for targeted therapy-based translational research. Front. Oncol..

[104] Thibault B., Castells M., Delord J.P., Couderc B. (2014). Ovarian cancer microenvironment: Implications for cancer dissemination and chemoresistance acquisition. Cancer Metastasis Rev..

[105] Nielsen S.R., Quaranta V., Linford A., Emeagi P., Rainer C., Santos A., Ireland L., Sakai T., Sakai K., Kim Y.S. (2016). Macrophage-secreted granulin supports pancreatic cancer metastasis by inducing liver fibrosis. Nat. Cell Biol..

[106] O’Connell J.T., Sugimoto H., Cooke V.G., MacDonald B.A., Mehta A.I., LeBleu V.S., Dewar R., Rocha R.M., Brentani R.R., Resnick M.B. (2011). VEGF-A and Tenascin-C produced by S100A4+ stromal cells are important for metastatic colonization. Proc. Natl. Acad. Sci. USA.

[107] Curtis M., Kenny H.A., Ashcroft B., Mukherjee A., Johnson A., Zhang Y., Helou Y., Batlle R., Liu X., Gutierrez N. (2019). Fibroblasts Mobilize Tumor Cell Glycogen to Promote Proliferation and Metastasis. Cell Metab..

[108] Nieman K.M., Kenny H.A., Penicka C.V., Ladanyi A., Buell-Gutbrod R., Zillhardt M.R., Romero I.L., Carey M.S., Mills G.B., Hotamisligil G.S. (2011). Adipocytes promote ovarian cancer metastasis and provide energy for rapid tumor growth. Nat. Med..

[109] Lehtonen A., Himanen P., Saraste M., Niittymaki K., Marniemi J. (1986). Double-blind comparison of the effects of long-term treatment with doxazosin or atenolol on serum lipoproteins. Br. J. Clin. Pharmacol..

[110] Miranda F., Mannion D., Liu S., Zheng Y., Mangala L.S., Redondo C., Herrero-Gonzalez S., Xu R., Taylor C., Chedom D.F. (2016). Salt-Inducible Kinase 2 Couples Ovarian Cancer Cell Metabolism with Survival at the Adipocyte-Rich Metastatic Niche. Cancer Cell.

[111] Tang J., Pulliam N., Ozes A., Buechlein A., Ding N., Keer H., Rusch D., O’Hagan H., Stack M.S., Nephew K.P. (2018). Epigenetic Targeting of Adipocytes Inhibits High-Grade Serous Ovarian Cancer Cell Migration and Invasion. Mol. Cancer Res..

[112] Schumann T., Adhikary T., Wortmann A., Finkernagel F., Lieber S., Schnitzer E., Legrand N., Schober Y., Nockher W.A., Toth P.M. (2015). Deregulation of PPARbeta/delta target genes in tumor-associated macrophages by fatty acid ligands in the ovarian cancer microenvironment. Oncotarget.

[113] Gerber S.A., Rybalko V.Y., Bigelow C.E., Lugade A.A., Foster T.H., Frelinger J.G., Lord E.M. (2006). Preferential attachment of peritoneal tumor metastases to omental immune aggregates and possible role of a unique vascular microenvironment in metastatic survival and growth. Am. J. Pathol..

[114] Clark R., Krishnan V., Schoof M., Rodriguez I., Theriault B., Chekmareva M., Rinker-Schaeffer C. (2013). Milky spots promote ovarian cancer metastatic colonization of peritoneal adipose in experimental models. Am. J. Pathol..

[115] Zhang Q.X., Magovern C.J., Mack C.A., Budenbender K.T., Ko W., Rosengart T.K. (1997). Vascular endothelial growth factor is the major angiogenic factor in omentum: Mechanism of the omentum-mediated angiogenesis. J. Surg. Res..

